# The effect of coffee/caffeine on postoperative ileus following elective colorectal surgery: a meta-analysis of randomized controlled trials

**DOI:** 10.1007/s00384-021-04086-3

**Published:** 2022-01-06

**Authors:** Tzu-Wei Yang, Chi-Chih Wang, Wen-Wei Sung, Wen-Chien Ting, Chun-Che Lin, Ming-Chang Tsai

**Affiliations:** 1 School of Medicine and Institute of Medicine, Chung Shan Medical University, Taichung, 402 Taiwan; 2 Division of Gastroenterology and Hepatology, Department of Internal Medicine, Chung Shan Medical University Hospital, Taichung, 402 Taiwan; 3 Department of Urology, Chung Shan Medical University Hospital, Taichung, 402 Taiwan; 4 Division of Colon and Rectum, Department of Surgery, Chung Shan Medical University Hospital, Taichung, 402 Taiwan; 5 Department of Internal Medicine, China Medical University Hospital, Taichung, 404 Taiwan; 6 School of Medicine, China Medical University, Taichung, 404 Taiwan

**Keywords:** Postoperative ileus, Coffee, Caffeine, Colorectal surgery, Colectomy

## Abstract

**Purpose:**

Postoperative ileus (POI) is the most common complication of elective colon resection. Coffee or caffeine has been reported to be useful in improving gastrointestinal function after abdominal surgery. This study aimed to investigate the effect of coffee/caffeine on POI in patients undergoing elective colorectal surgery.

**Methods:**

We searched Cochrane library, Embase, PubMed, and ClinicalTrials.gov (until July 2021) to identify randomized controlled trials (RCTs) evaluating the effect of coffee or caffeine on bowel movements and POI in patients undergoing elective colorectal surgery. The mean difference (MD) for continuous outcomes and risk ratio (RR) for dichotomous outcomes were calculated and are presented with 95% confidence intervals (CIs). A random effects model was used in all meta-analyses.

**Results:**

A total of four RCTs including 312 subjects met the inclusion criteria and were included in the meta-analysis. Postoperative coffee or caffeine consumption decreased the time to first bowel movement (MD, − 10.36 h; 95% CI, − 14.61 to − 6.11), shortened the length of hospital stay (MD, − 0.95 days; 95% CI, − 1.57 to − 0.34), and was associated with a decreased risk of the use of any laxatives after the procedure (RR, 0.64; 95% CI, 0.44 to 0.92). The time to first flatus, time to tolerance of solid food, risk of any postoperative complication, postoperative reinsertion of a nasogastric (NG) tube, and anastomotic leakage showed no statistical differences between groups.

**Conclusion:**

Postoperative coffee or caffeine consumption improved bowel movement and decreased the duration of hospital stay in patients undergoing elective colorectal surgery. This method is safe and can prevent or treat POI.

**Supplementary Information:**

The online version contains supplementary material available at 10.1007/s00384-021-04086-3.

## Introduction

Postoperative ileus (POI) is the most common complication of elective colon resection, with a complication rate of approximately 12% [1]. Several risk factors have been reported to be associated with POI including blood loss, advanced age, anastomotic leak, laparotomy approach, prolonged operative time, narcotic use, disseminated cancer, and respiratory comorbidities [2]. Many studies have investigated the pathophysiology involved in POI including an inflammatory response to intestinal trauma, increased inhibitory sympathetic activity, and inhibitory neurotransmitters in the intestinal tract [2, 3]. The prolonged symptoms include nausea, vomiting, abdominal distension, and intolerance to oral intake; these ultimately lead to a prolonged hospital stay, patient discomfort, and increased health care costs [4, 5].

Some non-pharmacological supplements and interventions have been reported to prevent POI, including the use of chewing gum, early enteral feeding, coffee, and acupuncture [2, 5, 6]. Coffee is a popular beverage, and evidence suggests that coffee consumption improves metabolic diseases, reduces some digestive malignancies (e.g., colon and liver cancer), and even decreases the risk of all-cause mortality [7–9]. Consuming coffee may also increase colon motility through the multifactorial effects of caffeine, polyphenols, dietary fiber, and Maillard reaction products and alter the gut-brain axis and gut microbiota [9–12]. The current evidence suggests that postoperative coffee or caffeine consumption may alleviate POI and improve gastrointestinal function after cesarean surgery and gynecological cancer surgery [13, 14].

Randomized control trials (RCTs) have reported conflicting results regarding the effects of postoperative coffee or caffeine consumption on POI among patients undergoing colorectal surgery [15–18]. Therefore, we conducted a systemic review and meta-analysis of RCTs that have used coffee or caffeine as a postoperative supplement to assess the potential benefits of coffee or caffeine in the recovery of gastrointestinal motility after colorectal surgery.

## Materials and methods

### Literature search strategy

In this study, we conducted a meta-analysis to evaluate the association between coffee/caffeine consumption and POI after colorectal surgery. The research was performed according to the Preferred Reporting Items for Systematic reviews and Meta-Analyses (PRISMA) principles [19]. Cochrane Central Register of Controlled Trials (CENTRAL), Embase, PubMed, and ClinicalTrials.gov were searched independently by 2 authors (T.W.Y. and C.C.W.) for relevant studies on July 6, 2021. Our search strategy is listed in Supplementary Table S1.

### Study selection criteria and data extraction

Determination of study eligibility and data extraction were performed independently by two reviewers (T.W.Y. and C.C.W.). The inclusion criteria were as follows: (1) RCT, (2) participants with benign or malignant colorectal disease who had undergone open or laparoscopic colectomy, and (3) study intervention involving coffee or caffeine supplementation after the procedure. The exclusion criteria were as follows: (1) abstract-only publications, (2) articles not written in English, and (3) no applicable endpoints. We extracted the data from the included studies and performed an intention-to-treat analysis. Otherwise, we used data that were available to use. For data reported as medians and interquartile ranges (IQRs), the mean and standard deviation (SD) were estimated according to the Cochrane Handbook for Systemic of Interventions [20]. The formulae are defined a $\mathrm{Mean}\approx \left(\mathrm{median}\right)$ s and $SD\approx \frac{q3-q1}{1.35}$. Another author (M.C.T.) confirmed the final determination.

### Outcome measures

The outcome measure was the improvement of POI. Our primary outcomes included the following: (1) time to first bowel movement, (2) time to first flatus, and (3) time to tolerance of solid food. The secondary outcomes were as follows: (1) length of hospital stay, (2) use of any laxative, (3) any postoperative complication, (4) postoperative reinsertion of a nasogastric (NG) tube, and (5) anastomotic leakage.

### Methodological quality

Two authors (T.W.Y. and C.C.W.) independently assessed all the included trials using Cochrane’s “Risk of Bias (RoB)” tool [21]. A third author (M.C.T.) confirmed the final determination after discussion. The potential bias was determined, and the following seven domains were included: random sequence generation, allocation concealment, blinding of participants and personnel, blinding of outcome assessment, incomplete outcome data, selective reporting, and other biases.

### Statistical analysis

We used Review Manager version 5.3 (RevMan for OS X; the Nordic Cochrane Centre, Copenhagen, Denmark) for data analysis. Continuous variables were calculated using the inverse variance method, and dichotomous variables were calculated using the Mantel–Haenszel method. A *P*-value of < 0.05 was considered statistically significant. Continuous outcomes are presented as the mean difference (MD), and dichotomous outcomes are presented as risk ratios (RRs) with 95% confidence intervals (CIs). A random effects model was used in all meta-analyses. We assessed the heterogeneity by using the *I*^2^ test developed by Higgins [22].

## Results

### Characteristics of the included studies

**Fig. 1:**
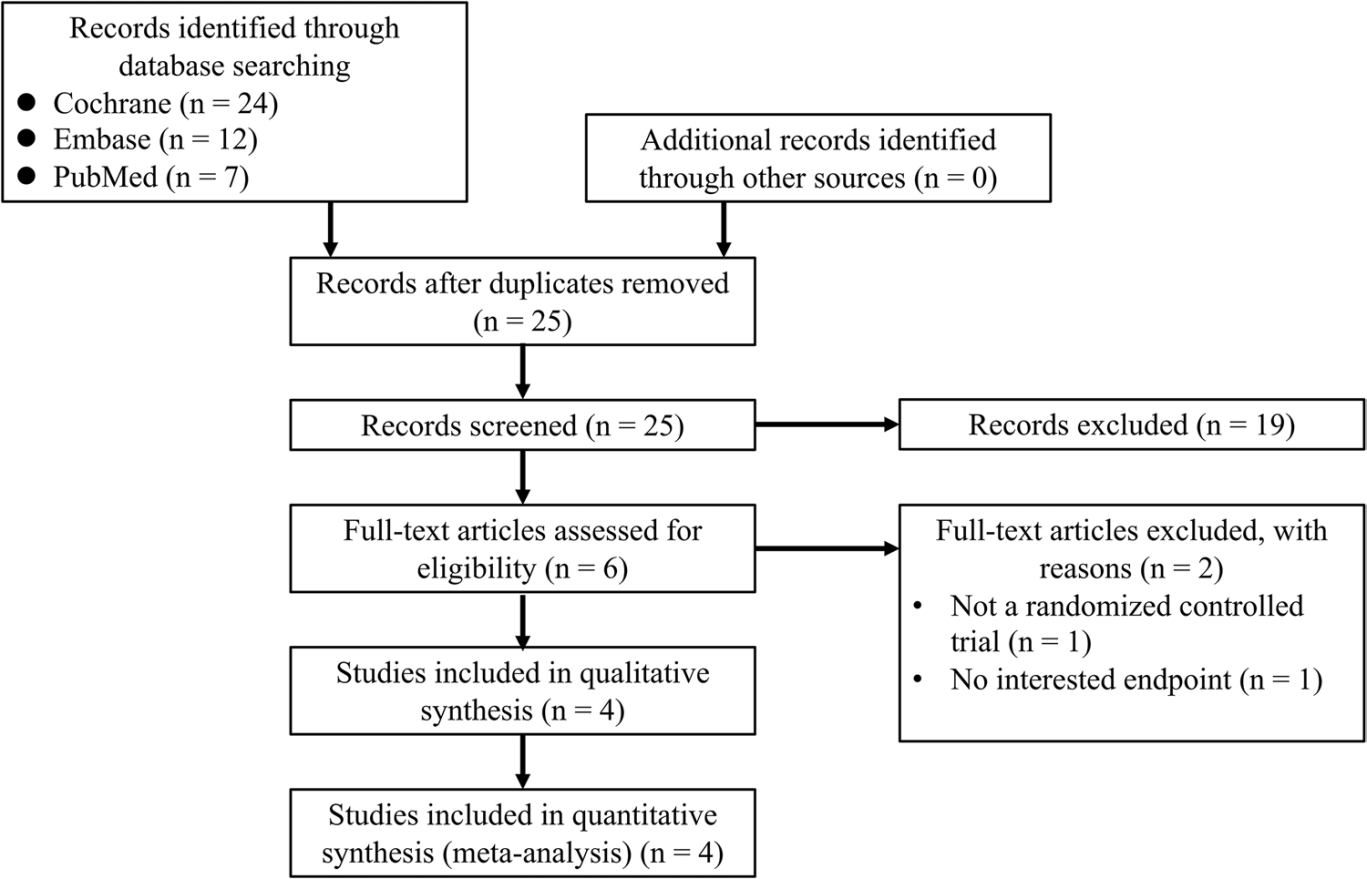
PRISMA study flow diagram

Our primary literature search identified a total of 43 studies. Among them, four RCTs with 312 participants met the inclusion criteria and were included in the meta-analysis. The PRISMA flow diagram is presented in Fig. 1. The protocol for this review has been registered in the PROSPERO network (registration number: CRD42021289459). The characteristics of the included studies are shown in Table 1. All the procedures in the studies were elective, and most of them were performed via a laparoscopic approach; however, 48 participants underwent open surgery, and five underwent conversion to open surgery. The outcomes showed no significant difference between open and laparoscopic colectomy in the original study [15]. The amount of coffee or caffeine used in these studies was near a standard cup of caffeinated coffee. Among the participants, 156 were included in the coffee or caffeine group (coffee [15–17] and caffeine [18]), and 156 were included in the control group (water [15, 16, 18] and tea without caffeine [17]) (Table 2). After randomization in these four studies, there was no statistically significant difference between groups in the number of cases of different surgical types.

**Table 1:** Characteristics of the included studies

Study	Year	Country	Study Design	Participants	Intervention*	Control*	Any postoperative complication (n/N)
Müller [15]	2012	Germany	Multicenter open-label RCT	Elective open or laparoscopic colonic resection for malignant or benign diseases (*n* = 79)	100 mL coffee 3 times daily	100 mL water 3 times daily	Intervention: (8/40) Control: (10/39)
Dulskas [16]	2015	Lithuania	Single-center, open-label RCT	Elective laparoscopic left-sided colonic resection for malignant diseases (*n* = 96)	100 mL coffee 3 times daily (group 1) 100 mL coffee without caffeine 3 times daily (group 2)	100 mL water 3 times daily (group 3)	Intervention: (1/30) Control: (1/30)
Hasler-Gehrer [17]	2019	Switzerland	Single-center, open-label RCT	Elective laparoscopic colon or rectal resection for benign or malignant disease (*n* = 115)	150 mL coffee 3 times daily	150 mL tea without caffeine 3 times daily	Intervention: (15/56) Control: (15/59)
Parnasa [18]	2021	Israel	Single-center, double-blinded RCT	Elective laparoscopic colon or rectal resection for either a malignant or a benign disease (*n* = 58)	100 mg of caffeine citrate in 50 mL of apple-flavored water 3 times daily	4 mL of water was diluted in 50 mL of apple-flavored water 3 times daily	Total (13/70)

^*^All the interventions or controls in the four studies were started on the first postoperative day

**Table 2:** Characteristics of the participants included in the studies

Study	Intervention	No. of participants	Mean age in years ± SD	Male/Female	Type of colonic disease (malignancy/benign)	Surgical approach (open/laparoscopic)	Operation time (range), min	Operative procedure, n (%)*
Müller [15]	Coffee	ITT: 40, PP: 35	62 ± 12	25/15	23/17	24/16	173 ± 56	Ileocecal resection: 10 (13), right hemicolectomy: 26 (33), left hemicolectomy: 9 (11), and sigmoid/rectosigmoid resection: 34 (43%)
	Water	ITT: 39, PP: 36	59 ± 15	19/20	22/17	24/15	183 ± 57	
Dulskas [16]	Coffee with caffeine	ITT: 32 PP:30	67.3 ± 6.8	16/14	30/0	0/30	102.0 ± 37.2	Anterior rectal resection with partial TME: 32 (36), left hemicolectomy: 17 (19), and sigmoid colectomy: 41 (46)
	Coffee without caffeine	ITT: 32 PP:30	62.4 ± 10.8	16/14	30/0	0/30	103.0 ± 42.5	
	Water	ITT: 32 PP:30	66.3 ± 9.1	16/14	30/0	0/30	98.0 ± 35.2	
Hasler-Gehrer [17]	Coffee	ITT: 56 PP:49	63 ± 12.6	31/25	23/33	1 (converted)/55	160 (136–185)	Ileocecal resection: 1 (1), Right hemicolectomy: 36 (31), Left hemicolectomy: 14 (12), Sigmoid resection: 60 (52), and Rectal resection: 4 (3)
	Tea without caffeine	ITT: 59 PP:47	69 ± 11.9	28/31	29/30	3 (converted)/56	150 (130–180)	
Parnasa [18]	Caffeine	ITT: 35 PP:30	56.90 ± 12.77	15/15	N/A	0/30	N/A	Right hemicolectomies (36.7), subtotal colectomies (17.8), and sigmoid/anterior resections (45.5)
	Water	ITT: 35 PP:28	55.36 ± 15.48	14/14	N/A	1 (converted)/27	N/A	

*ITT* intention-to-treat, *N/A* not available, *PP* per-protocol, *TME* total mesorectal excision

^*^The number of different surgical case types was not statistically significantly different between groups after randomization in the four studies

### Risk of bias of the included studies

**Fig. 2:**
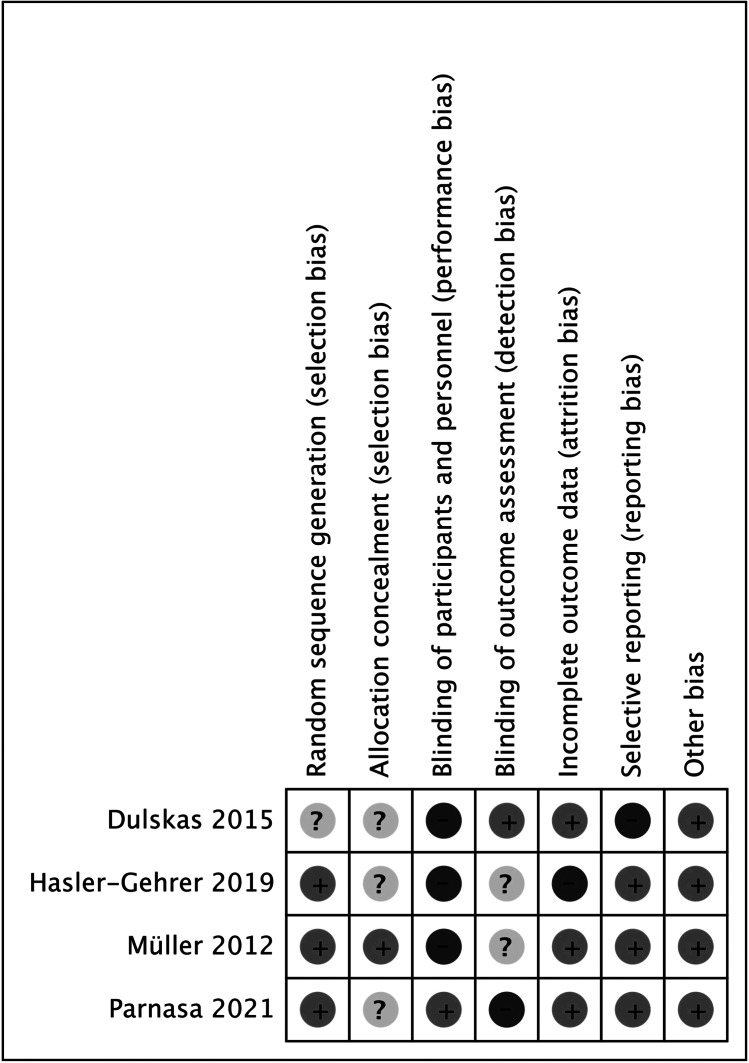
Risk of bias assessment of the included studies

The risk of bias is shown in Fig. 2. One study did not describe the method of randomization adequately [16]. Three studies were rated as having an unclear risk of bias on allocation concealment [16–18]. The difference in distribution of right- and left-sided hemicolectomy in between study groups might induce selection bias in one study [18]. Three studies designed as open-label trials were rated as having a high risk of performance bias [15–17]. The outcome was recorded by a blinded observer in only one study that was assessed as having a low risk of detection bias [16].

### Primary outcomes

**Fig. 3:**
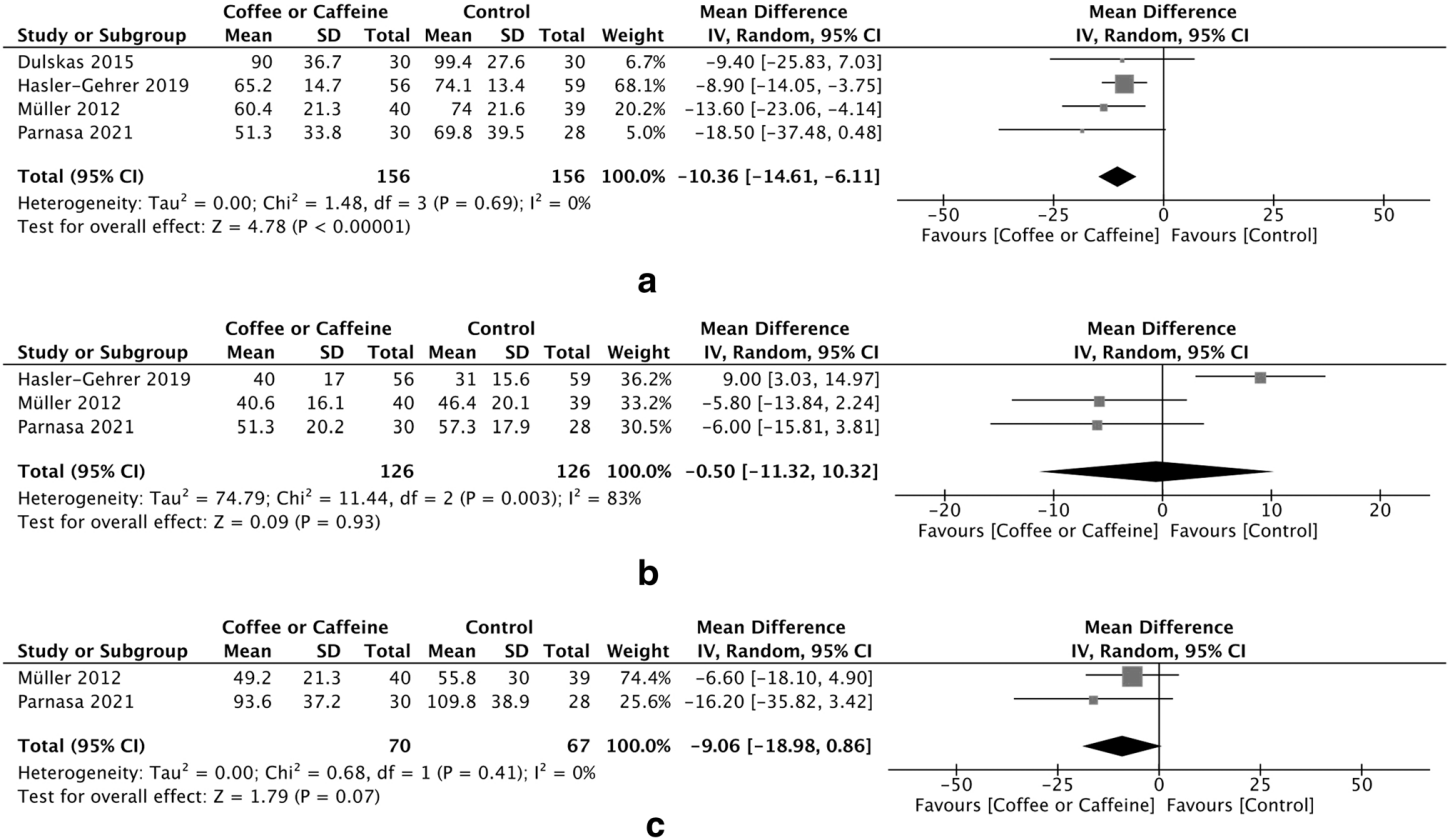
Forrest plot of the meta-analysis of the **A** time to first bowel movement, **B** time to first flatus, and **C** time to tolerance of solid food

The time to first bowel movement was reported in four studies (*n* = 312). The meta-analysis showed that postoperative coffee or caffeine consumption was associated with a significantly shorter time to first bowel movement, resulting in an MD of − 10.36 h (95% CI, − 14.61 to − 6.11 h; *P* < 0.00001) with no heterogeneity among the studies (*I*^2^ = 0%) (Fig. 3A).

The time to first flatus was reported in three studies (*n* = 252). The pooled data showed no difference in the time to first flatus between the coffee or caffeine group and the control group. The MD was − 0.5 h (95% CI, − 11.32 to 10.32 h; *P* = 0.93). There was remarkable heterogeneity across the included RCTs (*I*^2^ = 83%) (Fig. 3B).

The time to tolerance of solid food was reported in two studies (*n* = 137). There was no statistically significant difference in the time to tolerance of solid food between the coffee or caffeine group and the control group. The MD was − 9.06 h (95% CI, − 18.98 to 0.86 h; *P* = 0.07). There was no heterogeneity across the included RCTs (*I*^2^ = 0%) (Fig. 3C).

### Secondary outcomes

**Fig. 4:**
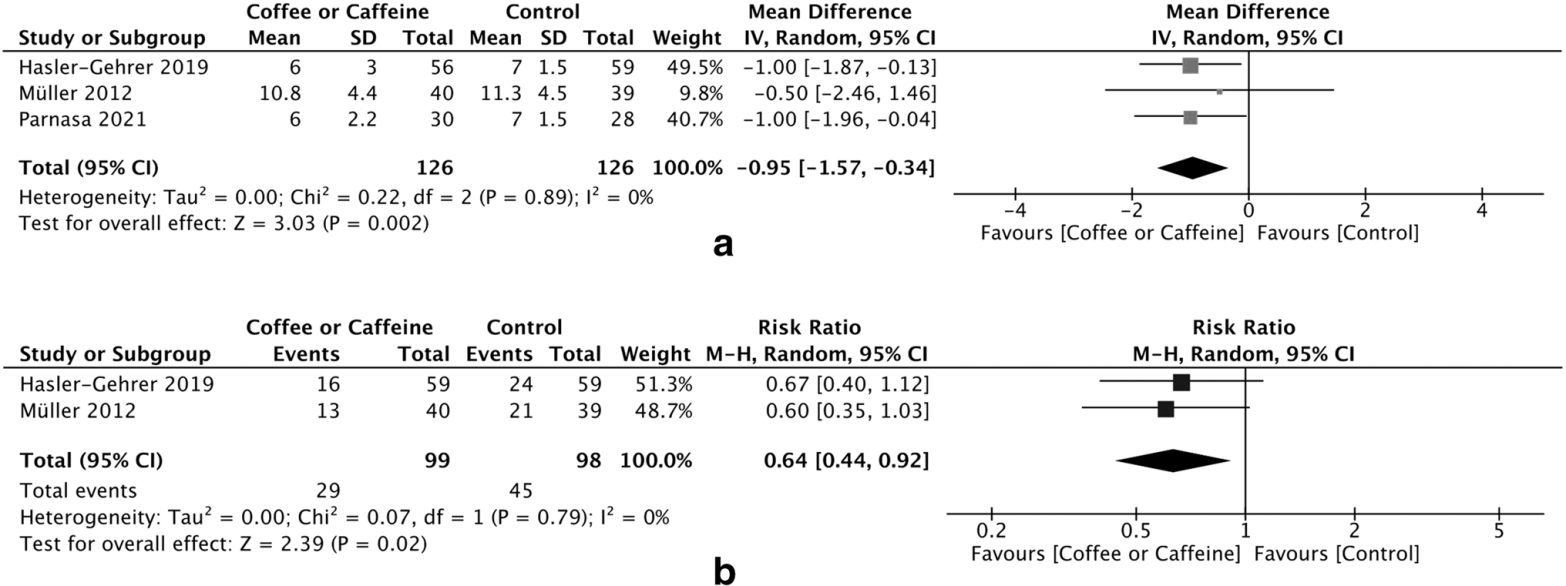
Forrest plot of the meta-analysis of the **A** length of hospital stay and **B** use of any laxative

Three studies reported the length of hospital stay (*n* = 252). Postoperative coffee or caffeine consumption was associated with a shorter length of hospital stay than the control, resulting in an MD of − 0.95 days (95% CI, − 1.57 to − 0.34 days; *P* = 0.002) with no heterogeneity among the studies (*I*^2^ = 0%) (Fig. 4A). Two studies reported the use of any laxatives after the procedure (*n* = 197). Postoperative coffee or caffeine consumption was associated with a lower risk of use of any laxatives after the procedure than the control. The RR was 0.64 (*I*^2^ = 0%, 95% CI, 0.44 to 0.92; *P* = 0.02) (Fig. 4B).

The pooled data from three studies (*n* = 254) showed no difference in the risk of any postoperative complication between the coffee or caffeine group and the control group (RR, 0.95; 95% CI, 0.58 to 1.54; *P* = 0.83; *I*^2^ = 0%) (Supplementary Fig. S1A). The pooled data from four studies (*n* = 312) showed no difference in the risk of postoperative reinsertion of an NG tube between the coffee or caffeine group and the control group (RR, 0.77; 95% CI, 0.37 to 1.58; *P* = 0.47; *I*^2^ = 0%) (Supplementary Fig. S1B). The pooled data from two studies (*n* = 194) revealed no difference in the risk of anastomotic leakage after the procedure between the coffee or caffeine group and the control group (RR, 0.48; 95% CI, 0.09 to 2.53; *P* = 0.39; *I*^2^ = 27%) (Supplementary Fig. S1C).

## Discussion

The meta-analysis of four RCTs aimed to evaluate the effect of postoperative coffee or caffeine consumption on the postoperative bowel recovery in patients undergoing elective colorectal surgery. The ability of coffee or caffeine to improve bowel movement and postoperative complications was investigated. The results showed that coffee or caffeine consumption improved the time to first bowel movement and decreased the length of hospital stay and the use of laxatives. The time to first flatus, time to tolerance of solid food, postoperative reinsertion of an NG tube, and presence of any postoperative complication including anastomotic leakage showed no statistically significant differences between the study groups.

An improved time to first bowel movement with postoperative coffee/caffeine consumption was found in all the included studies. The RCT by Müller et al. [15] enrolled patients who had received open or laparoscopic colectomy. The results showed similar improvements in bowel movement although open surgery is considered a risk factor for POI [23]. There was a trend toward shorter time to tolerance of solid food in the coffee/caffeine groups compared with the control groups; however, the difference was not statistically significant. The patients from the RCT by Hasler-Gehrer et al. [17] took solid food on the first postoperative day, and no obvious complications were reported. There was no difference in the presence of any postoperative complication including anastomotic leakage between the groups in these included studies. These findings are consistent with recent evidence suggesting that early enteral feeding is safe during patient recovery from colorectal surgery [2, 5].

Interestingly, Dulskas et al. [16] also reported that decaffeinated coffee was more effective than coffee with caffeine in shortening the time until the first bowel movement and the time until tolerance of solid food. Inconsistent with the previous report, either caffeinated coffee or decaffeinated coffee were associated with an increase in colonic motor activity [24, 25]. These findings were interpreted as some constituents other than caffeine affecting bowel movement. In addition, the chemical composition of coffee beans is very different and is severely affected by the roasting process (i.e., Maillard reaction), which produces newly formed contaminants [9]. These ingredients contained in coffee may have direct or indirect (via some molecules such as cholecystokinin, exorphins, gastrin, or motilin) effects on gut smooth muscle [9]. In addition, coffee induces secretions from the small intestine but is not associated with changes in small bowel transit [9]. One study used tea without caffeine as the control group [17]. Although a recent study found that yellow tea extract could improve loperamide-induced constipation in mice, the effect of tea on human gut motility is still largely unknown [26].

A strength of our meta-analysis is the inclusion of all RCTs specifically including patients who underwent colorectal surgery. The results suggest that the application of coffee/caffeine products after colorectal surgery could be an effective supplement to prevent or improve prolonged POI. The use of coffee/caffeine may not only improve patient discomfort but also decrease hospital stay and health care costs which could be considered as part of the enhanced recovery after surgery protocols.

Limitations of this study include the small sample size of the included RCTs and the fact that only four RCTs were available for analysis to date. There were limited data, and we were unable to perform subgroup analyses of patient characteristics and different surgical procedures, considering there may be overjudgment of the risk of selection and performance bias in the included RCTs which was assessed as high or unclear because it could not be blinded. Furthermore, the optimal coffee/caffeine dosage is unknown. Further studies are needed to determine the optimal dosage of coffee/caffeine and to investigate the effect of other chemical components such as polyphenols or melanoidins on the improvement in bowel movement.

## Conclusion

Coffee or caffeine products improve bowel movement and shorten the length of hospital stay. These products are safe and could be used as supplements in treating POI

## Supplementary Information

Below is the link to the electronic supplementary material.

Supplementary file1 (JPG 261 KB)

Supplementary file2 (JPG 299 KB)

Supplementary file3 (JPG 250 KB)

Supplementary file4 (DOCX 32 KB)
