# Supplementary figures and images for: The effect of coffee/caffeine on postoperative ileus following elective colorectal surgery: a meta-analysis of randomized controlled trials
Source: Int J Colorectal Dis. 2022 Jan 6;37(3):623–30. doi: 10.1007/s00384-021-04086-3 (PMC8885519; doi:10.1007/s00384-021-04086-3)

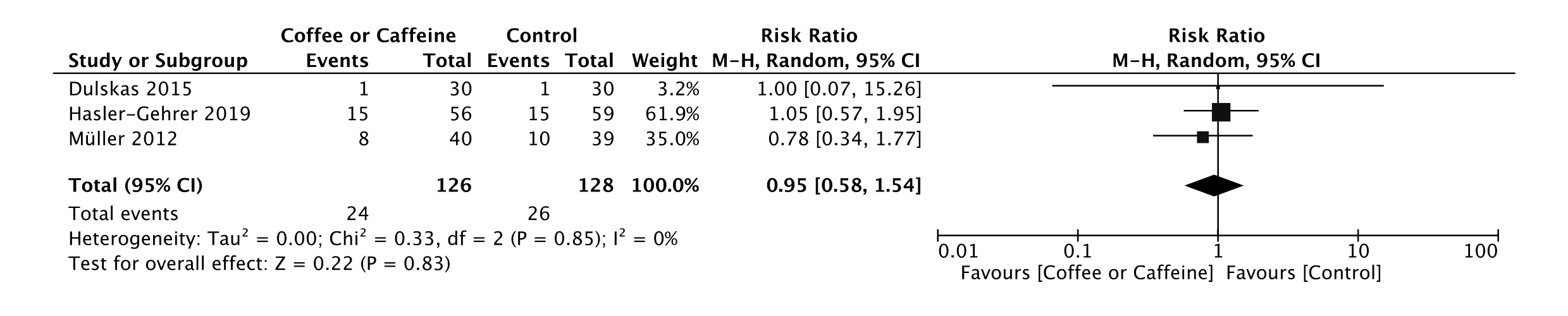

Supplement: Supplementary file 1 — Supplementary file1 (JPG 261 KB) [file 384_2021_4086_MOESM1_ESM.jpg]

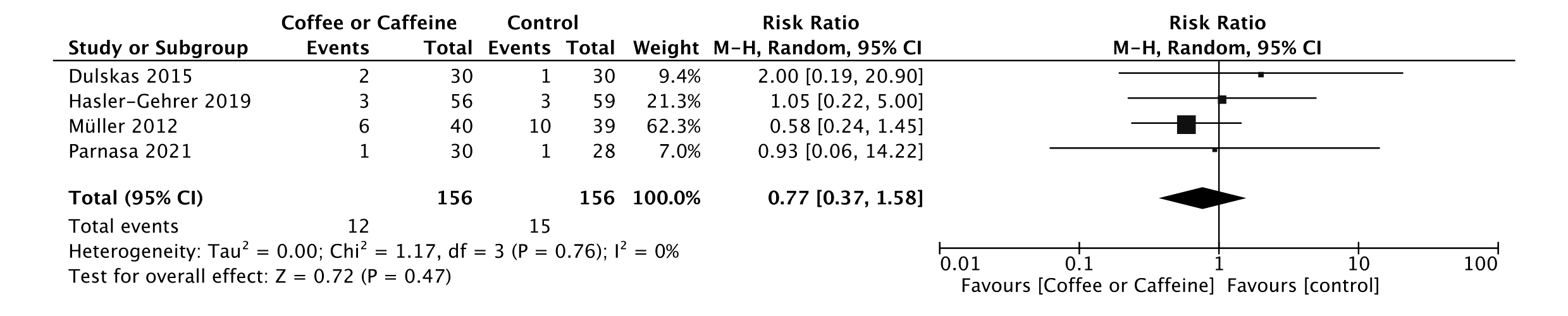

Supplement: Supplementary file 2 — Supplementary file2 (JPG 299 KB) [file 384_2021_4086_MOESM2_ESM.jpg]

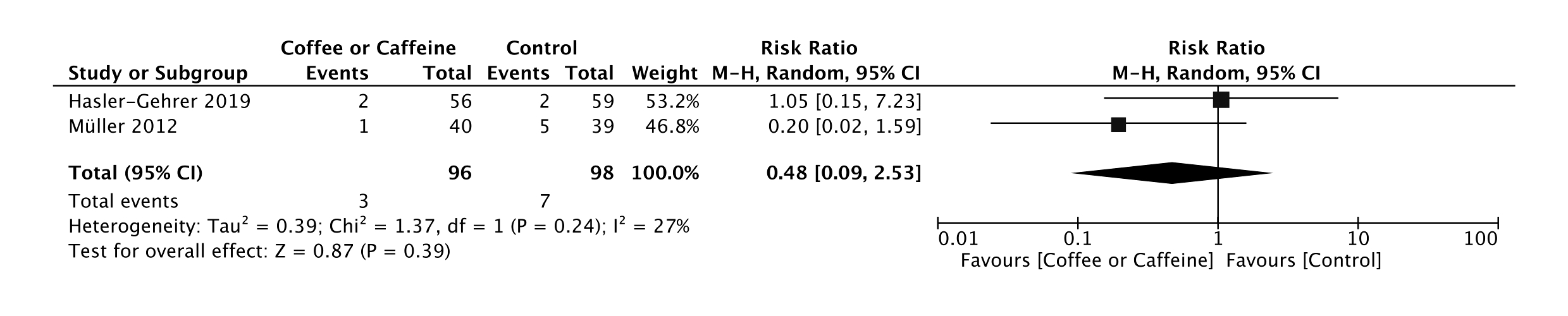

Supplement: Supplementary file 3 — Supplementary file3 (JPG 250 KB) [file 384_2021_4086_MOESM3_ESM.jpg]
